# Machine-Learned Codes from EHR Data Predict Hard Outcomes Better than Human-Assigned ICD Codes

**DOI:** 10.3390/make7020036

**Published:** 2025-04-17

**Authors:** Ying Yin, Yijun Shao, Phillip Ma, Qing Zeng-Treitler, Stuart J. Nelson

**Affiliations:** 1 Biomedical Informatics Center, George Washington University, Washington, DC 20052, USA; 2 Veterans Administration Hospital, Washington, DC 20422, USA

**Keywords:** machine learning prediction, ICD coding, impact factors, morbidity, mortality

## Abstract

We used machine learning (ML) to characterize 894,154 medical records of outpatient visits from the Veterans Administration Central Data Warehouse (VA CDW) by the likelihood of assignment of 200 International Classification of Diseases (ICD) code blocks. Using four different predictive models, we found the ML-derived predictions for the code blocks were consistently more effective in predicting death or 90-day rehospitalization than the assigned code block in the record. We reviewed records of ICD chapter assignments. The review revealed that the ML-predicted chapter assignments were consistently better than those humanly assigned. Impact factor analysis, a method of explanation of AI findings that was developed in our group, demonstrated little effect on any one assigned ICD code block but a marked impact on the ML-derived code blocks of kidney disease as well as several other morbidities. In this study, machine learning was much better than human code assignment at predicting the relatively rare outcomes of death or rehospitalization. Future work will address generalizability using other datasets, as well as addressing coding that is more nuanced than that of the categorization provided by code blocks.

## 1. Introduction

Electronic health records (EHRs) are widely used in the United States. As of 2021, the adoption rate reached 96% for non-federal acute care [1]. These systems not only enable patients and physicians to access clinical information electronically and streamline medical billing and claims processes, but they have also been recognized as valuable resources for assessing study feasibility and facilitating patient recruitment in clinical research. Beyond their clinical utility, EHRs also support observational studies in epidemiology, risk factor analysis, genomic association studies, and other areas [2,3].

A key initial step in utilizing EHR data for research involves phenotyping, the identification of patients with specific traits or medical conditions [4]. The most common data source for phenotyping is the International Classification of Diseases (ICD) codes. Developed by the World Health Organization, the ICD system was designed to standardize the classification and coding of diseases and has since been widely adopted by healthcare providers worldwide [5]. Accurate assignment of ICD codes is essential for precisely identifying and characterizing target populations in interventional and observational studies.

Typically, ICD codes are assigned by clinicians as part of their clinical practice or by trained medical coders who extract relevant information from clinical notes. However, this manual process is laborious and prone to error due to incomplete and disorganized documentation, limited resources (e.g., staffing and budget), human mistakes, and changes in coding standards [6]. Prior research has highlighted considerable variability and inconsistency in the assignment of ICD codes over time and across different clinical settings. For example, the transition from ICD-9 to ICD-10 can lead to substantial discrepancies in phenotype definitions [7–9]. Even within the same ICD version, inconsistencies in coding practices continue to be a significant challenge [10–12]. Such coding errors compromise subsequent clinical analyses by not accurately representing patient’s underlying conditions.

The recent emergence of machine learning (ML) has done much to improve and automate disease phenotyping using EHR data [13,14]. Compared with human coders, the advantages of the ML-based phenotyping methods include improved scalability for processing large volumes of data, enhanced consistency in applying phenotyping criteria, and the ability to detect complex patterns across multiple data types. However, most ML-based phenotyping methods typically require manual chart reviews by clinical experts to create “gold standards”, which is both time-consuming and tailored for specific research projects.

On the other hand, although the initial ICD codes assigned may not be perfect, they are not random. Patients with specific diagnosis codes often share common clinical characteristics—such as demographics, symptoms, and treatments—embedded within the EHR as structured (e.g., medication, procedures, etc.) or unstructured data (e.g., clinical notes). When treating the originally assigned ICD codes as a “silver standard”, ML can extract and capture these characteristics, creating a unique “fingerprint” of clinical features for each patient and generating ML-derived phenotypes. This methodology can potentially provide a more robust and consistent representation of patient conditions.

ICD codes are organized hierarchically, starting with broad disease and condition categories (chapters). These chapters are further subdivided into blocks, which group related conditions more specifically. In this study, we trained a ML model to create coarse-grained phenotypes, referred to as ML-derived ICD blocks, corresponding to ICD code blocks. To evaluate our hypothesis that ML-derived ICD blocks, which are more closely aligned with actual patient conditions, would improve the performance of downstream tasks such as predicting critical outcomes (mortality or hospitalization), we compared predictive performance using original ICD blocks from the EHR with that achieved using ML-derived ICD blocks across a range of ML models. By using the notes, procedures performed, and medications given to develop the ML models, we hoped to achieve a more detailed picture of each patient’s condition.

We chose to use the VA data for two reasons: The patient population tends to be relatively stable with fewer losses due to changes in providers or hospitals than in other datasets, and the data available include all the clinical notes, not generally available in other very large datasets. While predominantly male, our focus here was on testing whether ML predictions were more effective than human-assigned codes and considered that the differing population was unlikely to affect our results.

## 2. Materials and Methods

### 2.1. Data Procurement and Preparation

The data for this analysis were sampled from the Veterans Health Administration (VHA) Corporate Data Warehouse (CDW), which contains the full electronic health records of care provided to veterans by the VHA, across 18 Veterans Integrated Services Networks (VISNs). To ensure equal representation from each VISN, we randomly selected 9000 outpatient visits from each network between 2010 and 2019. Visits found to be lacking a valid diagnosis code or clinical note were then excluded. This resulted in a final dataset of 894,154 outpatient visits. This dataset was used to develop the ML-derived ICD model and to train and validate the patient outcome prediction models, as detailed below.

### 2.2. ICD Code Chapters and Code Blocks

The ICD-10 codebook is organized hierarchically into 22 chapters and subchapters (codes preceding the decimal point), referred to as code blocks. For example, Code D66 (Hereditary Factor VIII Deficiency) is the code block used for classic hemophilia. Given the limited on-site obstetrics and prenatal care services provided by the VHA, diagnosis code chapters related to these areas (O, P) were excluded. Additionally, chapters lacking meaningful diagnostic information (R, U–Z, representing symptoms and signs, causes of morbidity, and administrative codes) were omitted. Consequently, 16 code chapters (A–N, Q, S–T) and 200 ICD code blocks were included for data from 2016 to 2019. For the ICD-9-CM era (2010–2015), corresponding code chapters and blocks were determined using general equivalence mappings compiled by the Centers for Disease Control and Prevention.

### 2.3. Study Design

We first trained ML-derived ICD models using structured (e.g., demographics, medications) and unstructured (e.g., clinical notes) EHR data variables as predictors, with original ICD chapters/blocks as the target variable. Data from 2018 were employed for model training, validation, and testing. Subsequently, the trained model was then used to infer the ML-derived ICD scores for the selected outpatient visits from 2010 to 2017 and 2019.

**Figure 1:**
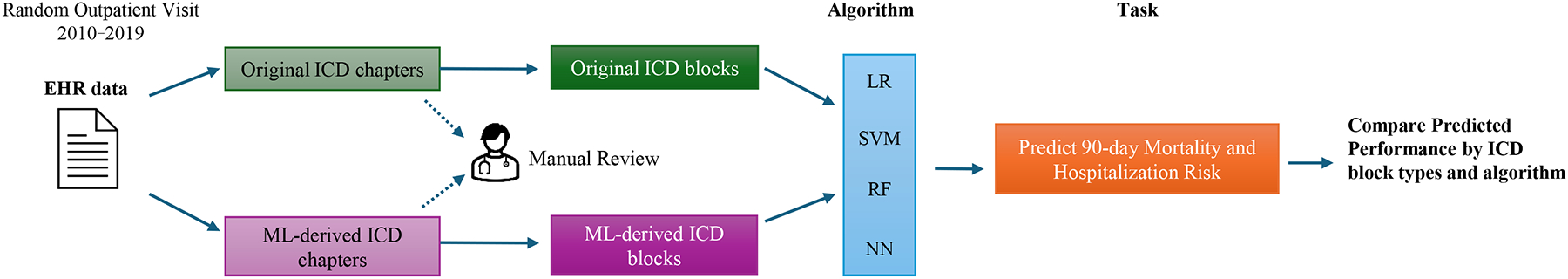
Study Design Abbreviation: LR = Logistic Regression; ML = Machine Learning; NN = Neural Network; RF = Random Forest; SVM = Support Vector Machine; VISNs = Veterans Integrated Services Networks

To investigate our central hypothesis that ML-derived ICD codes offer a more accurate and consistent representation of patient conditions than the original ICD codes, we conducted a series of experiments using a range of ML algorithms and feature sets to predict hard outcomes (90-day hospitalization or mortality). We compared the predictive performance when using the ML-derived ICD blocks as input features against that achieved with the original ICD blocks (Figure 1).

### 2.4. Machine Learning-Derived ICD Development

#### 2.4.1. Dataset

As described previously, a cohort of 162,000 outpatient visits from 2018 was selected. Visits lacking valid ICD diagnosis codes or clinical notes were excluded, resulting in a dataset of 116,434 outpatient visits. The demographic characteristics of the selected patients were as follows: 91% male, 71% White, and 20% African American, with a mean age of 63.6 years (SD = 15.2).

#### 2.4.2. Feature Extraction

Features were extracted from both structured and unstructured data sources. Structured features included medications dispensed within a 7-day window (grouped by VHA drug class) and procedures performed within a 7-day window (grouped by 3-digit Current Procedural Terminology [CPT] code). Clinic notes associated with the visit were also extracted for topic modeling.

#### 2.4.3. Topic Modeling

Latent Dirichlet allocation (LDA), an unsupervised machine learning technique for identifying latent topics within large text corpora, was employed to extract topic variables from the unstructured text data. The topic models were trained on 367,986 clinical notes from the selected 2018 visits. Each note underwent tokenization and preprocessing, including the removal of punctuation, standard stop words (e.g., “the”, “of”), domain-specific stop words (e.g., “patient”, “veteran”, “vet”), and low-frequency words occurring in fewer than 10 notes. LDA was then applied to the preprocessed notes with a specified number of 1000 topics. Theoretically, each topic is represented by a non-zero proportion in every note; however, in practice, topics with negligible proportions were considered absent. An empirical threshold was applied to define document–topic associations based on the virtual word count (calculated as the product of topic proportion and note word count). A topic was deemed present in the note if its virtual word count exceeded 4.

#### 2.4.4. Machine Learning Modeling

**Figure 2:**
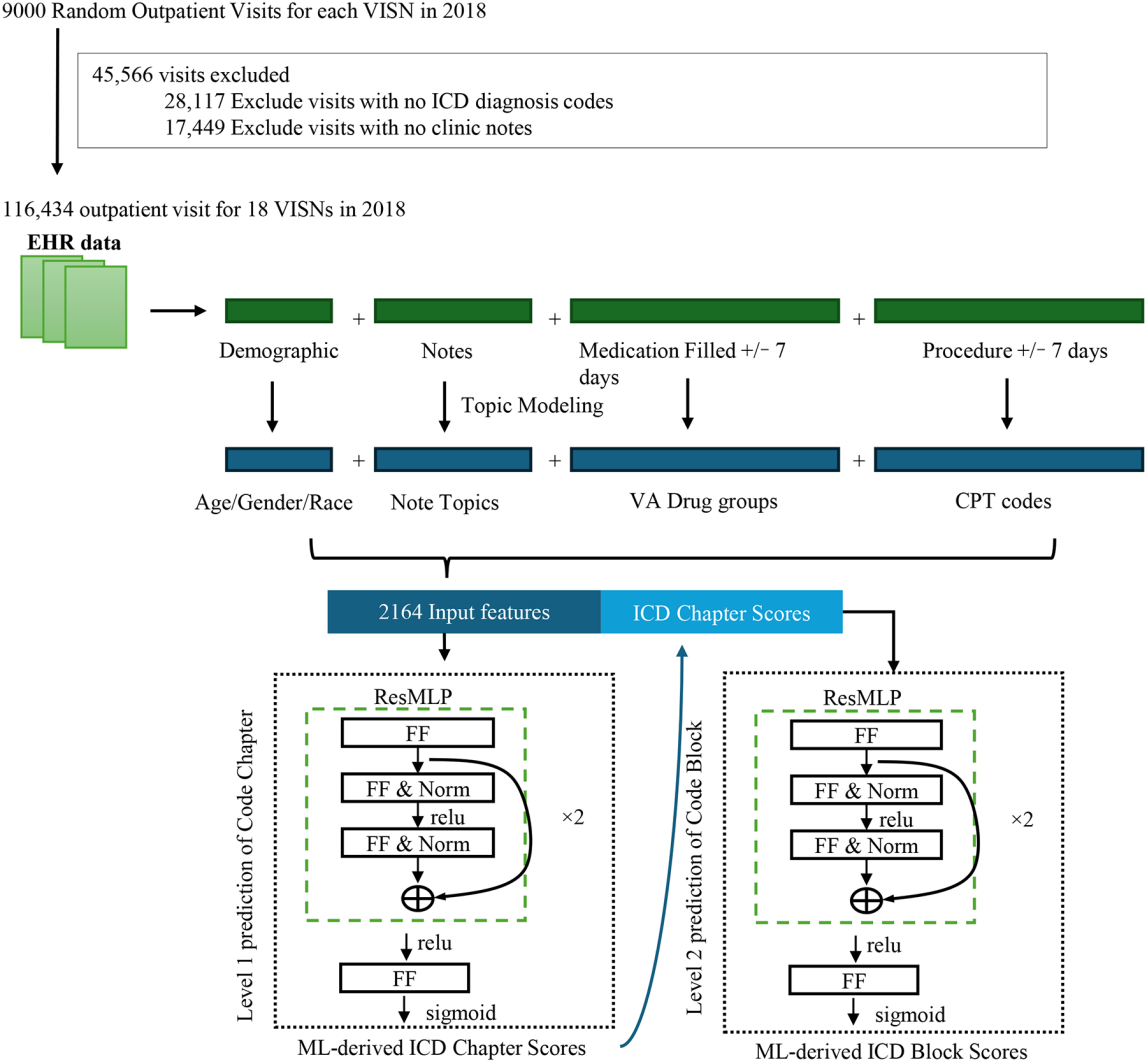
Deep Learning Model Architecture for Machine Learning-derived ICD Chapters/Blocks *Abbreviation: FF = Feedforward; ML = Machine Learning; Norm = Normalization; ReLu = Rectified Linear Unit;* ResMLP = Residual Multi-Layer Perceptron; *VISNs = Veterans Integrated Services Networks*

A two-level neural network model was designed to predict ICD block assignments, leveraging patient demographics and extracted note topics, medications, and procedures as input features. This resulted in a total of 2164 features (Figure 2). The model exploited the hierarchical structure of ICD codes: level 1 predicted the 16 ICD chapters, and level 2 predicted the ICD blocks. The model predicted both levels. Binary cross-entropy loss was minimized for both levels to optimize the model’s predictions.

The dataset was partitioned into training (64%), validation (16%), and test (20%) sets. Models were trained using stochastic gradient descent with a batch size of 256, the Adam optimizer, a learning rate of 0.0001, and a dropout rate of 0.25. The model with the highest micro-F1 score for predictions of level 2 (the ICD code blocks) was selected. Early stopping after 10 epochs without improvement was used to prevent overfitting. The final level 2 model generated multi-label predictions with probabilities for the 200 ICD blocks per visit, serving as the ML-derived ICD blocks. It is important to note that these ML-derived ICD blocks represent fuzzy classifications, with probability scores ranging from 0 to 1.

The developed topic model and neural network model for ICD block prediction were subsequently applied to other years (2010–2017, 2019).

### 2.5. Model Validation by Chart Review

To validate the neural network model’s performance in predicting ICD chapters, a manual review of 100 visits was conducted across three chapters: Infectious and Parasitic Diseases, Diseases of the Ear and Mastoid Process, and Diseases of the Respiratory System. These chapters were selected because they are relatively straightforward to predict and validate.

For each chapter, we identified two sets of 50 patient visits: one consisting of visits with the highest prediction scores for an ICD chapter but lacking the corresponding ICD code assignment and another with the lowest prediction scores but having the corresponding ICD code assigned. These visits represent cases of disagreement between the original ICD codes and the ML-derived ICD codes. The selected visits were then randomly shuffled, and a physician expert subsequently reviewed the clinical notes of these visits to determine the appropriate association of the diagnosis with the given code chapter. The expert’s assessments were considered the gold standard. Finally, we calculated the agreement between the gold standard, the original ICD chapter, and the ML-derived ICD chapters.

### 2.6. Outcome Prediction

#### 2.6.1. Dataset

A total of 116,434 outpatient visits from 2018 were utilized as the training and validation dataset, while 117,645 outpatient visits from 2019 served as the primary test dataset. Additionally, 659,776 remaining visits from 2010 to 2017 were employed for further validation.

#### 2.6.2. Independent Variables

The originally assigned ICD codes (both primary and secondary) were grouped into ICD code blocks. The presence or absence of 200 ICD code blocks, represented as binary features (1/0), served as one set of predictors. The ML-derived ICD scores, with probabilities represented as continuous variables, were assigned to each visit and used as the comparison set of predictors.

#### 2.6.3. Dependent Variables

The outcome of interest was 90-day hospitalization or death following an outpatient visit. This outcome was chosen for its independence from diagnosis codes while directly reflecting the patient’s condition.

#### 2.6.4. Modeling

A range of ML models were evaluated, including logistic regression, support vector machine (SVM), random forest, and a simple neural network (NN) with residual connections. Hyperparameters for the logistic regression, SVM, and random forest models were optimized using grid search with cross-validation, selecting the best parameter set based on the area under the receiver operating characteristic curve (AUC). The NN model was trained with a dropout rate of 0.25, a learning rate of 0.0001, a decay rate of 0.001, and stochastic gradient descent (SGD) as the optimizer. Model performance was assessed on the test set using accuracy, F1 score, recall, precision, and AUC.

#### 2.6.5. Impact Scores

To investigate why the outcome prediction results might occur, we compared the feature impacts on both DNN models using assigned and ML-derived code blocks for the outcome prediction. To do this, we calculated impact scores for each feature using a score mechanism previously developed [15]. A positive score value represents an increased positive impact of a feature on the outcome, with higher scores indicating a higher impact and vice versa.

### 2.7. Software

Topic modeling was conducted using MALLET (version 2.0) and a Java library (version 1.8). All other analyses were performed in Python (version 3.7), utilizing the scikit-learn (1.2.2), SciPy (1.8.0) , PyTorch (1.10.0), and Matplotlib (3.2.2) libraries for deep neural network development, data analysis, and data visualization.

## 3. Results

### 3.1. Manual Review of Original and Machine Learning-Derived ICD Chapters

The manual review consistently demonstrated better performance for the ML-derived ICD chapters than the original ICD chapters, which agreed with the gold standard. Results are shown in Table 1. For Diseases of the Ear and Mastoid Process, the ML-derived ICD chapters showed 68% agreement with the gold standard, while the original ICD chapters had only 32% agreement. The results were similar for Infectious and Parasitic Diseases (64% vs. 36%) and Diseases of the Respiratory System (58% vs. 42%).

### 3.2. Machine Learning-Derived ICD Blocks

Using the originally assigned ICD blocks as the “silver standard”, the model achieved an average AUC of 0.74 (SD = 0.15) across all 200 code blocks. A well-recognized challenge in predicting ICD codes, including code blocks, is the low prevalence of many codes. Notably, our model demonstrated strong performance for high-prevalence code blocks. As summarized in Table 2, the model achieved an average AUC of 0.92 (SD = 0.03) for the top 20 most common code blocks and 0.89 (SD = 0.05) for the top 50.

### 3.3. Outcome Prediction Comparisons

We compared the model’s performance in predicting 90-day hospitalization and mortality using original ICD or ML-derived ICD blocks. The ML-derived ICD blocks and ML classifiers were trained on 2018 data, and model performance was evaluated using 2019 data. Although performance varied across different machine learning algorithms, the neural network consistently performed best using the same feature sets. Based on AUC, recall, and F1 score, the best-performing classifier was the neural network using ML-derived ICD blocks (AUC = 0.7834, recall = 0.7320). As shown in Table 3, models utilizing ML-derived ICD consistently outperformed those using original ICD across different algorithms in terms of AUC.

We then examined the results across time and areas to see if the results were consistent. The improvement was consistent when the models were applied to data from previous years (2010–2017, Figure 3). The improvement was also present when the models were applied to each quarter in the years. Comparing the results across the 18 VISNs showed that the results were found consistently across the geographic regions. Furthermore, when using logistic regression with assigned ICD code blocks as the baseline, the improvement in AUC was more remarkable when comparing feature sets (ML-derived vs. assigned: 0.7588 vs. 0.7225) than when comparing machine learning algorithms (neural network vs. logistic regression: 0.7237 vs. 0.7225).

Table 4 compares 2 × 2 contingency tables for DNN model predictions using both assigned and ML-derived code blocks, with a prediction threshold of 0.5. As the outcome represents a relatively rare event, both models struggled to predict true positive events accurately. However, the model using ML-derived blocks achieved a substantially higher true positive rate (9%) compared to the model using assigned blocks (2%). This nearly five-fold improvement in true positive detection suggests that ML-derived blocks capture more nuanced data patterns relevant to outcome prediction.

### 3.4. Impact Factor Analysis

Concerned with what factors found by the ML models appeared to be more effective in prediction, we examined the impact factors for each. Impact factors for each human-assigned code block showed only small deviations from the expected value of 0. That is, none of the blocks appeared to have an important effect. However, with the ML-derived code blocks, some of the blocks had a much larger impact. The impact factors for the code blocks with the 20 highest impacts are shown in Table 5, with the impact factors for the assigned block for comparison.

## 4. Discussion

Although the difference in AUC may appear modest, a 6.1% improvement in the overall AUC is significant when one compares the differences in AUC with the various analytical methods. For example, the AUC difference between DNN and LR on the same dataset was only 0.1%. A closer examination using two classic 2 × 2 tables (Table 5) reveals a more pronounced impact. When comparing the results obtained using ML-derived ICD codes to those derived from assigned ICD codes, we observed that the ML-derived ICD codes demonstrated superior performance in predicting adverse hard outcomes such as death or rehospitalization.

While observing that kidney disease has predictive value in the VA population is not surprising, as patients with kidney diseases are more likely to be hospitalized due to multiple comorbidities and the need for complex management, the strength of the predictive power was unanticipated. It is important to note that the ML code blocks have a fuzzy score ranging from 0 to 1. Thus, they not only code the presence/absence of a disease but also potentially its severity, which makes them more sensitive in predicting hospitalization and death. What is surprising is the relative position of kidney disease versus other candidates, such as cardiovascular disease or respiratory disease, for our “hard outcome” prediction. This finding stimulates a variety of clinical questions and will require further investigation.

ICD codes are extensively used in clinical and research settings, serving as the backbone for phenotyping, billing, and outcome prediction efforts. However, the existing data quality issues associated with ICD code assignments are often overlooked in clinical research [7], with potentially critical consequences. Errors in coding can propagate downstream, impacting analyses and modeling outcomes. The reliance on questionable or inconsistent ICD code assignments raises significant concerns about the reliability of predictions derived from such data.

Past efforts to address these challenges, such as phenotyping initiatives, have frequently relied heavily on ICD codes or focused narrowly, limiting their generalizability [13,14]. In this study, we developed an ML model approach to predict a comprehensive spectrum of ICD blocks over time and across different VA healthcare facilities. This approach leverages the rich information available within the VHA CDW to mitigate the limitations of individual ICD codes. A manual review of the ML-derived ICD assignments demonstrated high agreement with independent clinician assessments, validating the accuracy and reliability of our method. Importantly, our findings show that ML-derived ICD blocks consistently outperformed original ICD blocks in predicting critical outcomes such as all-cause mortality and hospitalization.

This approach carries significant implications. By leveraging the power of ML, we can create more robust and accurate representations of patient conditions. Moreover, the flexibility of our ML model enables training and adaptation on diverse datasets, making it applicable to a wide range of healthcare settings and research questions.

Several potential explanations account for these findings. First, errors in medical diagnosis may reflect clinicians’ thinking at the time of coding, which could inadvertently contribute to poorer patient outcomes. Clinicians operating under time constraints may lack sufficient familiarity with coding practices and rules, leading to errors in diagnosis code assignments. Furthermore, a failure of “representational semantic integrity”, where multiple coding options may exist for a single condition, can contribute to misassignments.

Despite these advances, our study has certain limitations. Firstly, the training and testing of our ML model were conducted on a subset of VHA data and focused exclusively on outpatient populations. Additionally, by restricting our analysis to the prediction of ICD code blocks and their associated outcomes, our work was confined to a categorical level. Moreover, we evaluated only two hard outcomes—mortality and hospitalization—which, while significant, do not capture the full spectrum of clinical endpoints that could benefit from this approach.

Future research should address these limitations by expanding to other EHR datasets and exploring additional outcomes. Broader application across diverse populations and settings could further validate and refine the generalizability of our findings. Additionally, investigating more nuanced disease categorizations and integrating richer contextual information could enhance predictive accuracy and applicability in clinical practice.

## Figures and Tables

**Figure 3:**
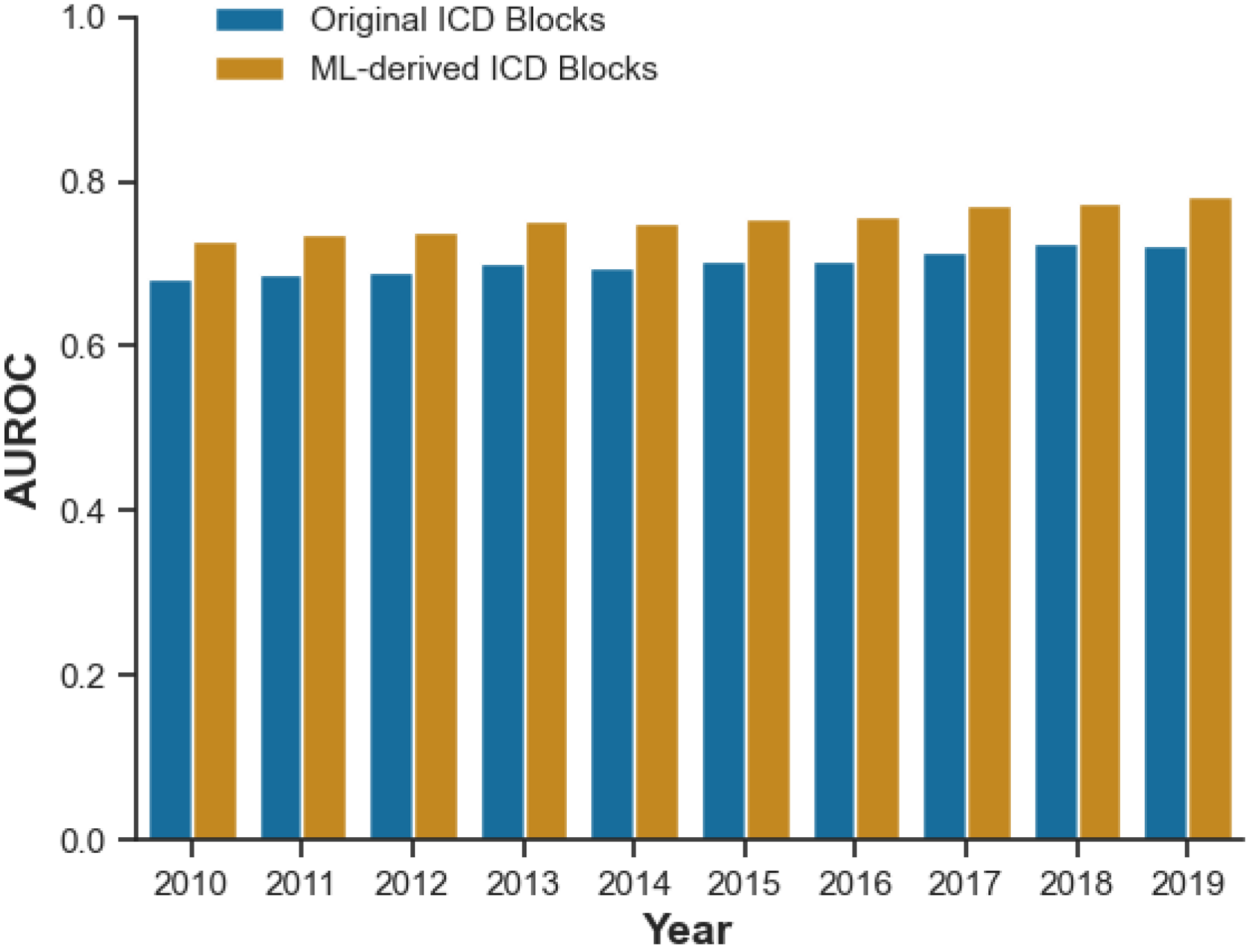
Comparison of Model Performance: Original vs. Machine Learning-Derived ICD Blocks in Predicting 90-Day Risk of Hospitalization and Mortality Using the Neural Network by Year

**Figure 4:**
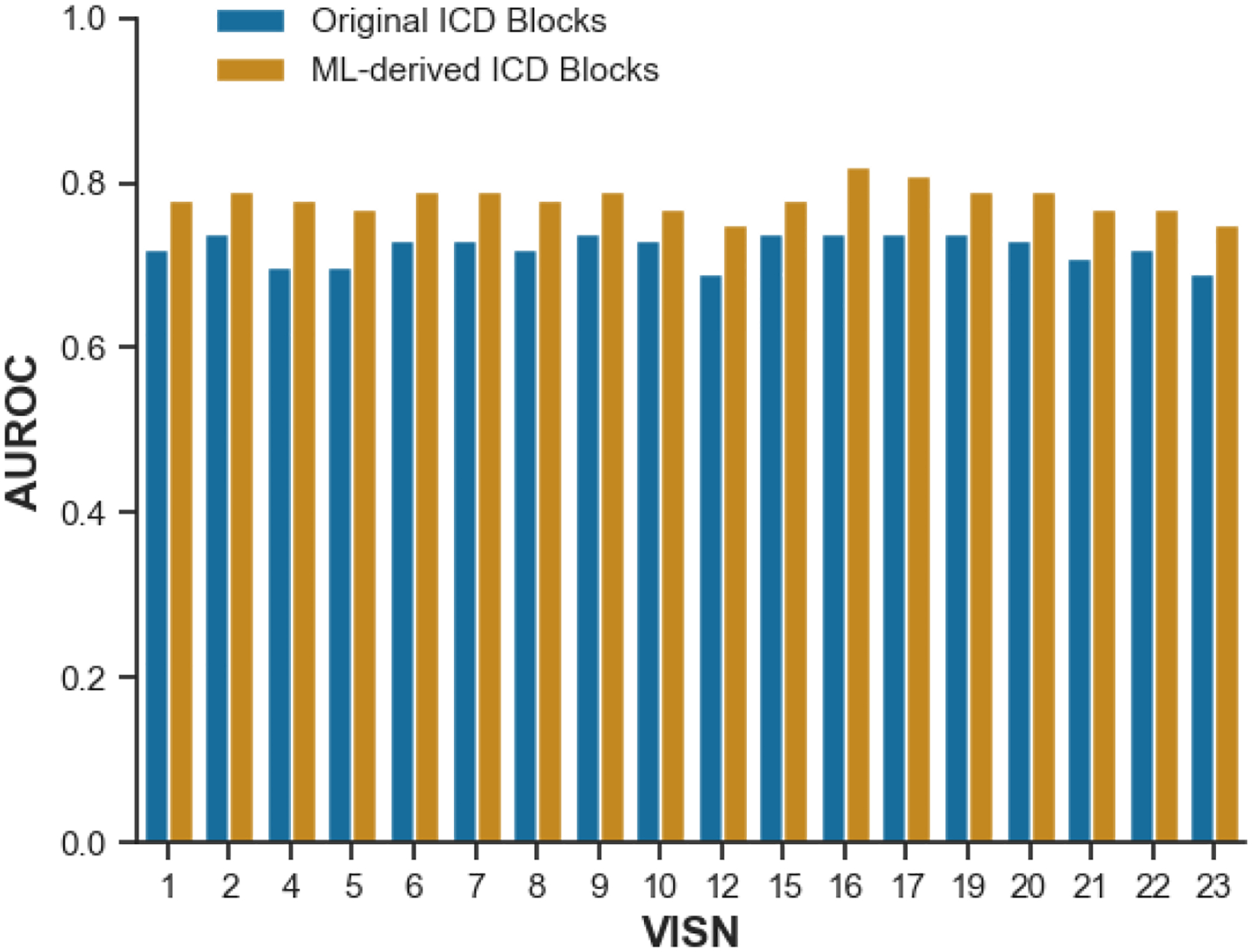
Comparison of Model Performance: Original vs. Machine Learning-Derived ICD Blocks in Predicting 90-Day Risk of Hospitalization and Mortality Using the Neural Network by VISN in 2019

**Table 1:** Comparison of Agreement Between Original ICD Chapter or Machine Learning-derived ICD Chapter, and Manual Reviews

	Original Chapter	ML-derived Chapter
Code Chapter	Review Agreement	Review Agreement
Infectious and parasitic disease	42.00%	58.00%
Diseases of the ear and mastoid process	32.00%	68.00%
Diseases of the respiratory system	36.00%	64.00%

**Table 2.** Model performance for machine learning-derived ICD blocks.

	AUC
All 200 Blocks, Mean ± SD	0.7409 (± 0.1543)
Top 10 prevalent Blocks, Mean ± SD	0.9248 (±0.0210)
Top 20 prevalent Blocks, Mean ± SD	0.9214 (± 0.0298)
Top 50 prevalent Blocks, Mean ± SD	0.8935 (± 0.0529)

**Table 3:** Performance Comparison of Classifiers Predicting 90-Day All-Cause Mortality or Hospitalization Risk Using Original ICD Blocks and ML-reassigned ICD Blocks in 2019

Classifier	Features	Recall	F1-Score	Precision	Accuracy	AUC
LR	Original Blocks	0.687	0.315	0.205	0.663	0.722
	ML-derived Blocks	0.702	0.342	0.226	0.695	0.759
SVM	Original Blocks	0.664	0.317	0.208	0.677	0.723
	ML-derived Blocks	0.701	0.344	0.228	0.698	0.751
RF	Original Blocks	0.651	0.312	0.205	0.675	0.707
	ML-derived Blocks	0.713	0.332	0.217	0.676	0.7510
NN	Original Blocks	0.638	0.323	0.216	0.697	0.724
	ML-derived Blocks	0.732	0.363	0.241	0.709	0.783

**Table 4:** Comparison of 2×2 Tables for Outcome Prediction Using DNN Models with Assigned or ML-derived ICD Blocks

	Outcome	
**Using Assigned ICD Blocks**	**TRUE**	**FALSE**
**Positive**	**253**	**160**
**Negative**	**13080**	**104452**

**Using ML-derived ICD Blocks**	**TRUE**	**FALSE**
**Positive**	**1207**	**975**
**Negative**	**12126**	**103637**

**Table 5.** Impact Scores for Top 20 ML-Derived Blocks with highest impact

Code Block Range	Description	Impact Scores for the DNN models	
		Assigned Blocks	ML-Derived Blocks
N17–N19	Acute kidney failure and chronic kidney disease	0.754	9.452
D60–D64	Aplastic and other anemias and other bone marrow failure syndromes	0.386	7.529
F30–F39	Mood [affective] disorders	0.110	5.890
G89–G95	Other disorders of the nervous system	0.185	5.183
F10–F19	Mental and behavioral disorders due to psychoactive substance use	0.842	5.131
D00–D09	In situ neoplasms	0.420	4.326
F01–F09	Mental disorders due to known physiological conditions	0.607	4.078
T80–T88	Complications of surgical and medical care, not elsewhere classified	0.541	4.018
I40–I4A	Chronic lower respiratory diseases	0.240	3.970
H15–H22	Disorders of sclera, cornea, iris and ciliary body	−0.079	3.777
G30–G32	Other degenerative diseases of the nervous system	0.361	3.555
S00–S09	Injuries to the head	−0.072	3.535
C78	Secondary neuroendocrine tumors	−0.207	3.458
N30–N39	Other diseases of the urinary system	0.335	3.344
I30–I5A	Other forms of heart disease	0.470	3.307
I60–I69	Cerebrovascular diseases	0.451	3.191
C64–68	Malignant neoplasms of urinary tract	0.638	3.168
H30–H36	Disorders of choroid and retina	−0.193	3.148
C7A	Malignant neuroendocrine tumors	−0.059	3.072
Q00–Q02	Congenital malformations of the nervous system	0.402	2.970

## Abbreviations

EHR: Electronic health record
ICD: International Classification of Diseases
VACDW: Veterans Central Data Warehouse
VHA: Veterans Health Administration
VISN: Veterans Integrated Services Network
ML: Machine learning
DNN: Deep neural network
LR: Logistic regression
RF: Random forest
SVM: Support vector machine
LDA: Latent Dirichlet allocation
FF: Feed forward
Norm: Normalization
ReLu: Rectified linear unit
ResMLP: Residual multi-layer perceptron
NN: Neural network
AUC: Area under the curve
SCD: Stochastic gradient descent
